# Trabecular meshwork ultrastructural changes in primary and secondary glaucoma

**DOI:** 10.1038/s41598-024-83834-1

**Published:** 2025-01-02

**Authors:** Aparna Rao, Tirupathi Rao, Nagapriya Banka, Sirisha Senthil, Saumya Jaketi

**Affiliations:** 1 Glaucoma Service, LV Prasad Eye Institute, Kallam Anji Reddy Campus, Hyderabad, 500034 India; 2 Ophthalmic Pathology Laboratory, L V Prasad Eye Institute, Kallam Anji Reddy Campus, 500034 Hyderabad, India; 3 Prof Krothapalli Ophthalmic Research Biorepository, L V Prasad Eye Institute, 500034 Hyderabad, India

**Keywords:** Trabecular meshwork, Ultrastructure, Scanning electron microscope, Primary glaucoma, Secondary glaucoma, Diseases, Medical research

## Abstract

To examine ultrastructural changes in the trabecular meshwork (TM) in patients with primary and secondary glaucoma using scanning electron microscopy (SEM). This was a qualitative descriptive hospital-based study on the ultrastructure of the TM. Pure TM samples were collected after microincisional trabeculectomy from 26 patients with primary or secondary glaucoma and 10 control samples from eye bank donor corneas. SEM was used to analyze structural changes in the TM beams, corneoscleral meshwork (CSM), and juxtacanalicular (JCT) regions. Morphological features were compared between groups and correlated with histopathological findings. SEM revealed flattened and broadened TM beams in the JCT, resembling controls, often with a dumbbell configuration. Histopathological examination (HPE) and SEM showed rounded TM beams with considerable thinning, especially in primary angle-closure glaucoma (PACG), compared to primary open-angle (POAG) and pseudoexfoliation glaucoma (XFG). Maximum thinning in all primary glaucoma occurred in the CSM region, with minimal changes in the JCT region despite a reduction in cellularity in both regions. In steroid glaucoma, amorphous, glistening material was found on the TM beams in the JCT and CSM. XFG eyes displayed vesicular bodies adjacent to fibrillar material scattered diffusely over the TM beams, particularly in the CSM, differing from the platelet clumps seen in regular blood clots. TM beam thinning in primary glaucoma primarily affects the CSM region, sparing the JCT region. Amorphous deposits or vesicular bodies, seen only in steroid-induced glaucoma and XFG, suggest different mechanisms of TM damage in these glaucoma types.

**Supplementary Information:**

The online version contains supplementary material available at 10.1038/s41598-024-83834-1.

## Introduction

A key structure in regulating aqueous humor outflow and maintaining IOP is the trabecular meshwork (TM), a specialized tissue located in the anterior chamber angle of the eye^1,2^. Dysfunction in the TM’s ability to drain aqueous humor effectively leads to increased intraocular pressure (IOP), a major risk factor for the development of glaucoma. The trabecular meshwork consists of multiple layers of interconnected trabeculae, which filter and control the outflow of aqueous humor^1^. It is composed of endothelial cells, collagen fibers, and extracellular matrix (ECM), and the structural integrity and cellular function of these components are crucial in maintaining proper aqueous outflow^1–3^. Distinct histopathological changes in the trabecular meshwork are reported in different types of glaucoma, including primary open-angle glaucoma (POAG), primary angle-closure glaucoma (PACG), pseudoexfoliation glaucoma (PXG) and steroid-induced glaucoma (SIG)^2,4^. While secondary forms of glaucoma are reported to cause more structural and functional damage to the TM tissue, most studies have reported the differences seen in postmortem tissues^2,4–9^. While excessive accumulation of fibrillar, Glycosaminoglycans (GAGs), or extracellular material between thinned trabecular beams is a prominent feature in XFG, SIG, and POAG, respectively, the collapse of the TM beams are characteristics of PACG^4,6^. While thinning of the TM beams with reduced cellularity is reported to be more prominent in the JCT region in glaucoma and aging tissues, specific changes in the different regions that determine the phenotype in POAG, PACG, XFG, or secondary glaucoma are unknown. While it may be possible that TM dysfunction may be a singular point of convergence by various insults in primary and secondary glaucoma, it may be logical to expect differential changes in the different regions subserving different functions in the TM. Most studies report findings from surgical ex-vivo trabeculectomy specimens, which often include portions of sclera or cornea from patients with varying disease severity or from postmortem tissues collected after death^2,3,5,7–9^. Microincisional trabeculectomy (MIT) ensures the procurement of pure TM tissue without sclera/corneal contamination and preserves architecture that allows the study of regional structural differences in patients^10–12^. This pilot study evaluates the TM ultrastructural features in SEM and HPE of TM tissues harvested by MIT in patients with severity-matched primary and secondary glaucoma.

## Methods

The TM specimens were obtained from patients with POAG, PACG, XFG, or SIG undergoing glaucoma surgery at a tertiary eye care in south India from January 2024 to June 2024 for uncontrolled IOP. An informed written consent was obtained from all subjects and/or their legal guardian(s). Age-matched control TM samples were sourced from donor corneoscleral buttons retrieved 3–6 h after death from the institutional eye bank (Table S1). POAG or PACG included patients > 40 years with uncontrolled IOP > 22 mm Hg on anti-glaucoma medications, open or closed angles respectively, with corresponding disc/visual field glaucomatous damage and visually significant cataract. Pseudoexfoliation glaucoma was diagnosed in adult patients with IOP > 22 mm Hg, the presence of exfoliative material in the eye, and visual field/disc damage. Steroid-induced glaucoma (SIG) was diagnosed in patients with IOP greater than 22 mm Hg following the use of topical steroids for any reason, accompanied by open angles, no anterior chamber inflammation, and evidence of optic disc and visual field damage. Patients with no vision, neovascular glaucoma, retinal associations, or neurodegenerative disorders, were excluded. The experimental protocol was approved by the institutional review board of LV Prasad Eye Institute (EC-035) and adhered to the tenets of the Declaration of Helsinki.

All statistical analysis was dne using stata corp (version 12, USA). For sample size calculation, we used apriori data on TM cellularity in earlier studies.^1,5–7^ To detect an effect size or difference of 0.2 in TM cellularity (cellularity defined as the ratio of the number of trabecular cell nuclei counted per solid tissue area in studies) between normal and other glaucoma types and 90% power of the study and alpha error set at 0.05, we calculated atleast 3 samples of each control and each glaucoma type.^7^

### TM tissue harvesting

The minimally invasive trabeculectomy (MIT) procedure is described in detail elsewhere^10,11^. In brief, a goniotomy is performed using an MVR blade through a temporal corneal incision. Straight vitreoretinal scissors are used to make two radial incisions at the upper edge of the trabecular meshwork over 3–5 clock hours in the nasal quadrant. The edge of the trabecular meshwork thus delineated is then grasped with 25-gauge vitreoretinal end-gripping forceps and gently stripped away in one motion. The TM tissues were flattened over a filter paper and then sent for SEM analysis in vials containing glutaraldehyde. Some tissues were cut into halves with one half sent for HPE in formalin-filled vials while the second half was sent in glutaraldehyde for SEM. Care was taken to flatten the tissue with the scleral side placed down on the filter paper (and the intracameral side facing up).

For controls, the TM strips were procured from cadaver donor corneoscleral tissue (*n* = 10, procured within 4–6 h of death) in a similar technique as above, taking care to dissect only the TM from the donor tissue.

### SEM and histopathological analysis protocol

For SEM, the TM samples were washed with 1xPBS (Phosphate buffer saline), fixed with 2.5% Glutaraldehyde – for 24 h at 4°C, and rinsed with distilled water before 2% Osmium tetra Oxide for 2 h, followed by dehydration through graded ethanol (10%, 30%, 50%, 70%, 90% and 100% for 30 min each) and HMDS (hexamethyldisilane) for 30 min and finally air dried overnight. Then, the tissue was sputtered with gold palladium for 90 s using a high vacuum evaporator (SC7620 Quorum Sputter Coater.) and visualized using a scanning electron microscope (SEM) (Carl Zeiss-Model EVO 18, Carl Zeiss, Germany).

The voltage used for acquiring the SEM images ranged between 5 and 20 kV.

For histopathological analysis, the formalin-fixed tissues were processed in an automated tissue processor TP 1020 (Leica Biosystems, Nussloch, Germany) and embedded in paraffin perpendicularly to ensure representative sections of each TM layer when cut into sections, Fig. S1. They were further stained with hematoxylin and eosin stain for examination under light microscope (BX 51, Olympus. The images were obtained using Magcam MU2A 2MP ½ CMOS Sensor digital Microscope Camera mounted on Olympus CX43 microscope.

The morphological features of the TM beams, the intertrabecular spaces in different regions, cellularity in different regions, and any additional qualitative features were compared between the controls and glaucoma TM tissues. Correlation with the histopathological features was done in selected cases where the tissues were adequate for splitting for SEM and HPE.

## Results

We obtained TM from 26 patients (17males, 9 females) with glaucoma who underwent MIT in the stated period, that included 11POAG, 3 PACG, 8 XFG, and 4 steroid glaucoma with a mean age of 64 ± 14.4 years (32–83 years). Concurrent HPE and SEM analysis from the same TM tissue were possible in 17 of 26 patients. We also procured control TM from 10 donor corneoscleral buttons from the eye bank with a mean age of 65 ± 8.7 years. Table 1 and Table S1 present the clinical details of the patients and control samples whose TM were analysed.

**Table 1:** Clinical characteristics of patients whose trabecular meshwork procured by microincisional trabeculectomy were analysed using scanning electron microscopy.

Variable	Mean ± standard deviation
Age (years)	64 ± 14.4
Gender	19 males 7 females
Diagnosis	11POAG, 3 PACG, 8 XFG 4 SIG
Mean deviation (dB)	-15 ± 5.6
Mean IOP at the time of surgery (mm Hg)	28 ± 12.8

Primary open-angle glaucoma-POAG, primary angle-closure glaucoma -PACG, pseudoexfoliation glaucoma -PXG, steroid-induced glaucoma-SIG.

### General features in controls and glaucoma cases

**Fig. 1:**
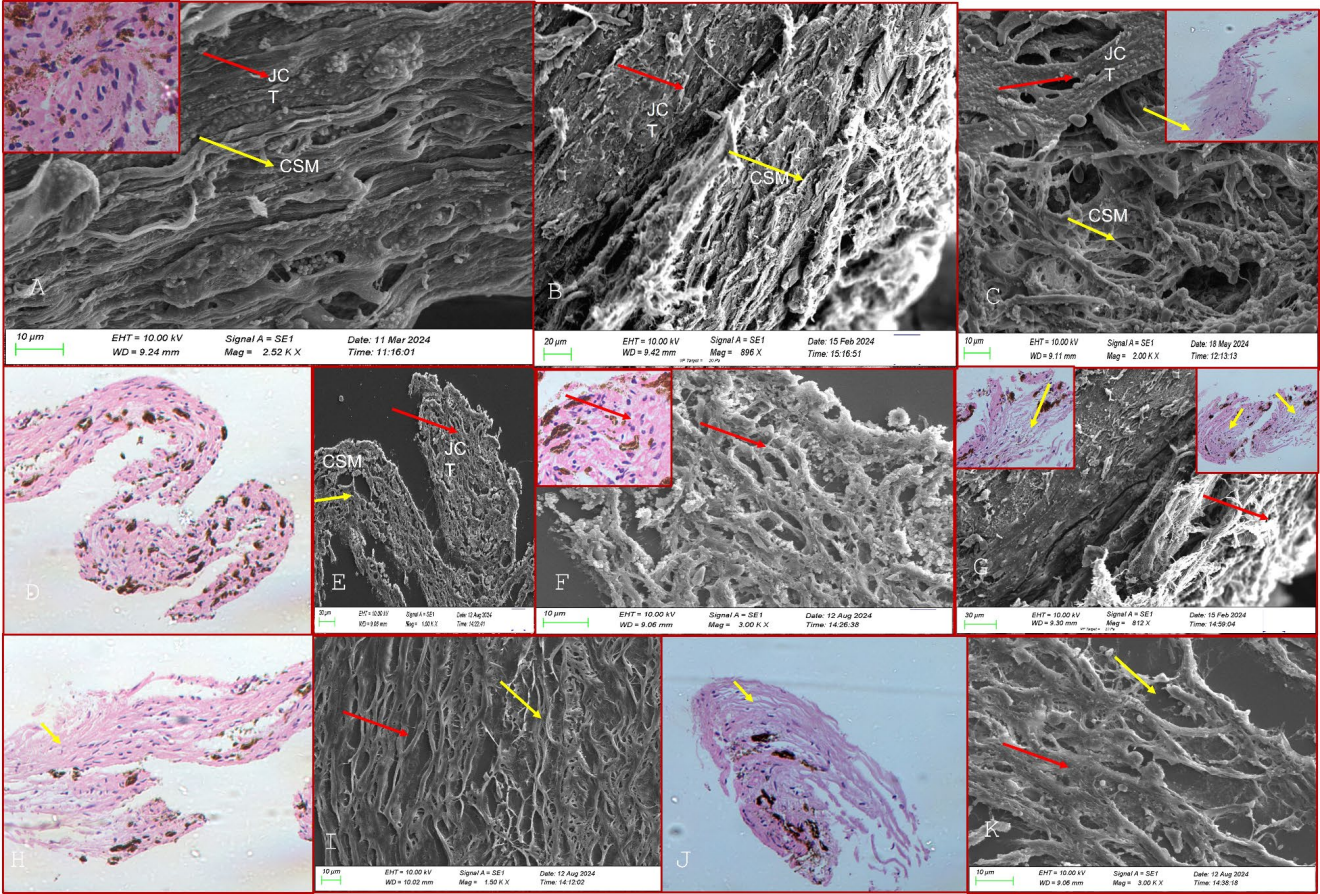
(**A**–**K**) show the histopathological (HP) and scanning electron microscopic (SEM) features of trabecular meshwork harvested from control donor corneoscleral buttons. The TM beams in** A**–**K** are broad and flat in the juxtacanalicular (JCT) region (red arrows) and corneoscleral region (yellow arrows) with numerous nucleated cells on histopathological examination, HPE, (**D**, **H**,** J**, insets in **A**, **C**, **F**, **G**). The JCT region is seen to have arrow intertrabecular spaces than the CSM region both on HPE and SEM sections (A-K-see text for full description).

**Fig. 2:**
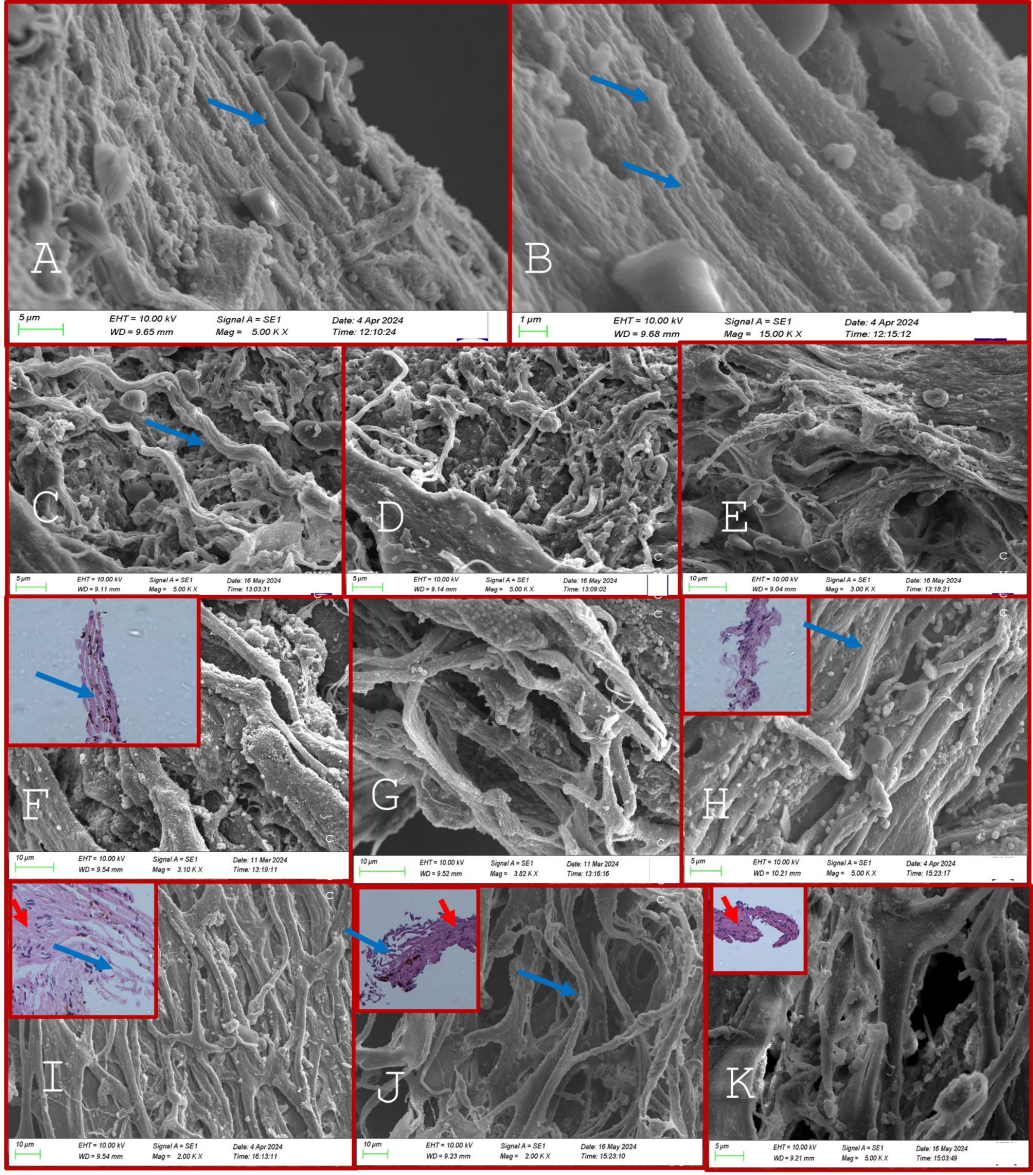
(**A**, **B**) show the TM beams in controls while (**C**–**K**) shows the HP and SEM features in the trabecular meshwork harvested after microincisional trabeculectomy in patients with primary open-angle glaucoma. The TM beams (blue arrows) in the CSM regions are thinner and irregularly arranged compared to controls (**A**,** B** -see full text for full description) with reduced cellularity on HP sections (insets **F**, **H**, **I**, **J**, **K**). **J** shows similar thinned TM beams in the CSM region seen on SEM and HP (inset) with those in the JCT region beams appearing normal despite reduced cellularity (K and inset).

**Fig. 3:**
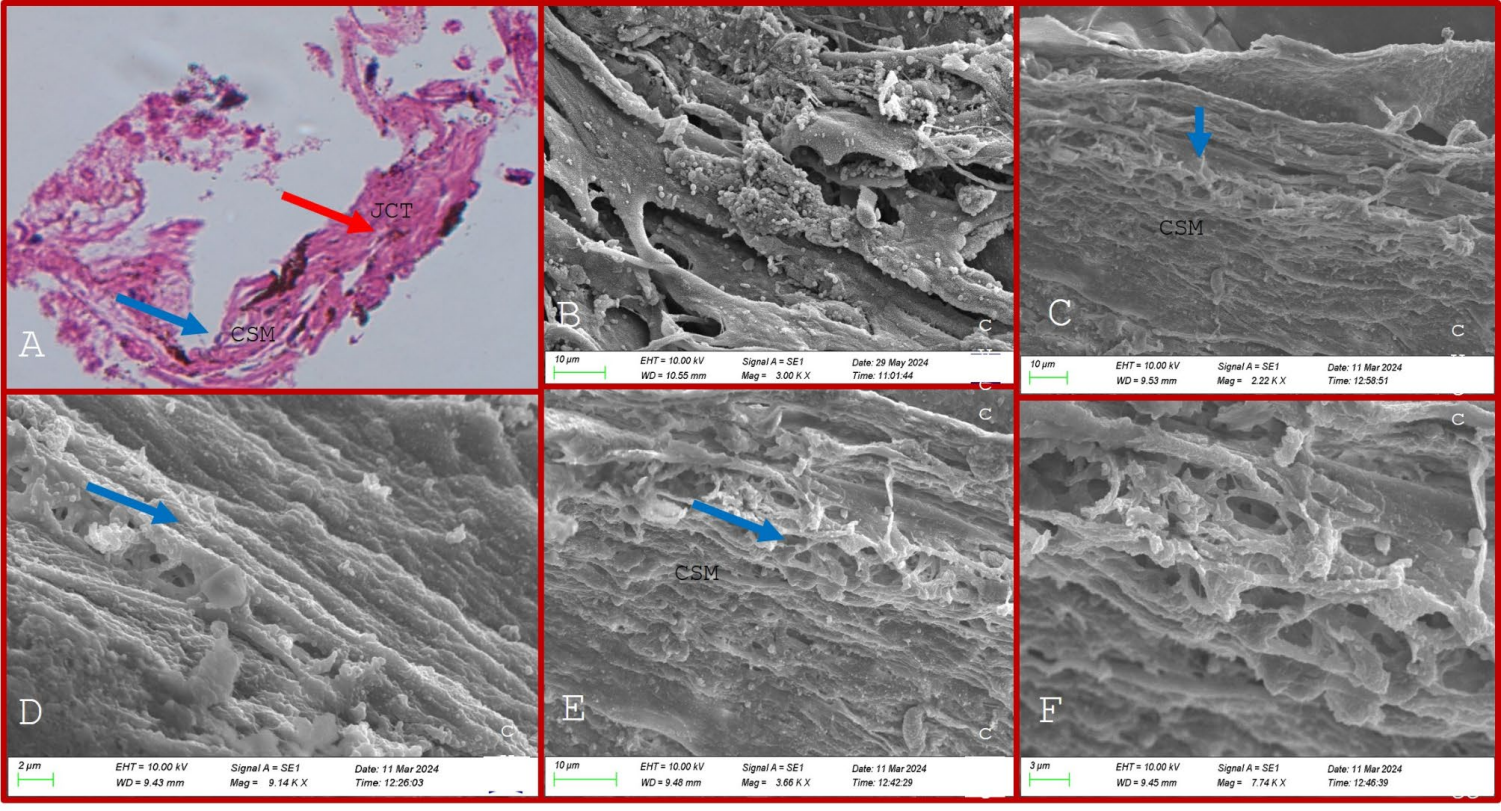
(**A**–**F**) shows the HP and SEM features in primary angle closure glaucoma, (**A**) shows maximally thinned irregular beams (red arrows) in the CSM regions on HP which is also confirmed on SEM (**B**–**F**).

**Fig. 4:**
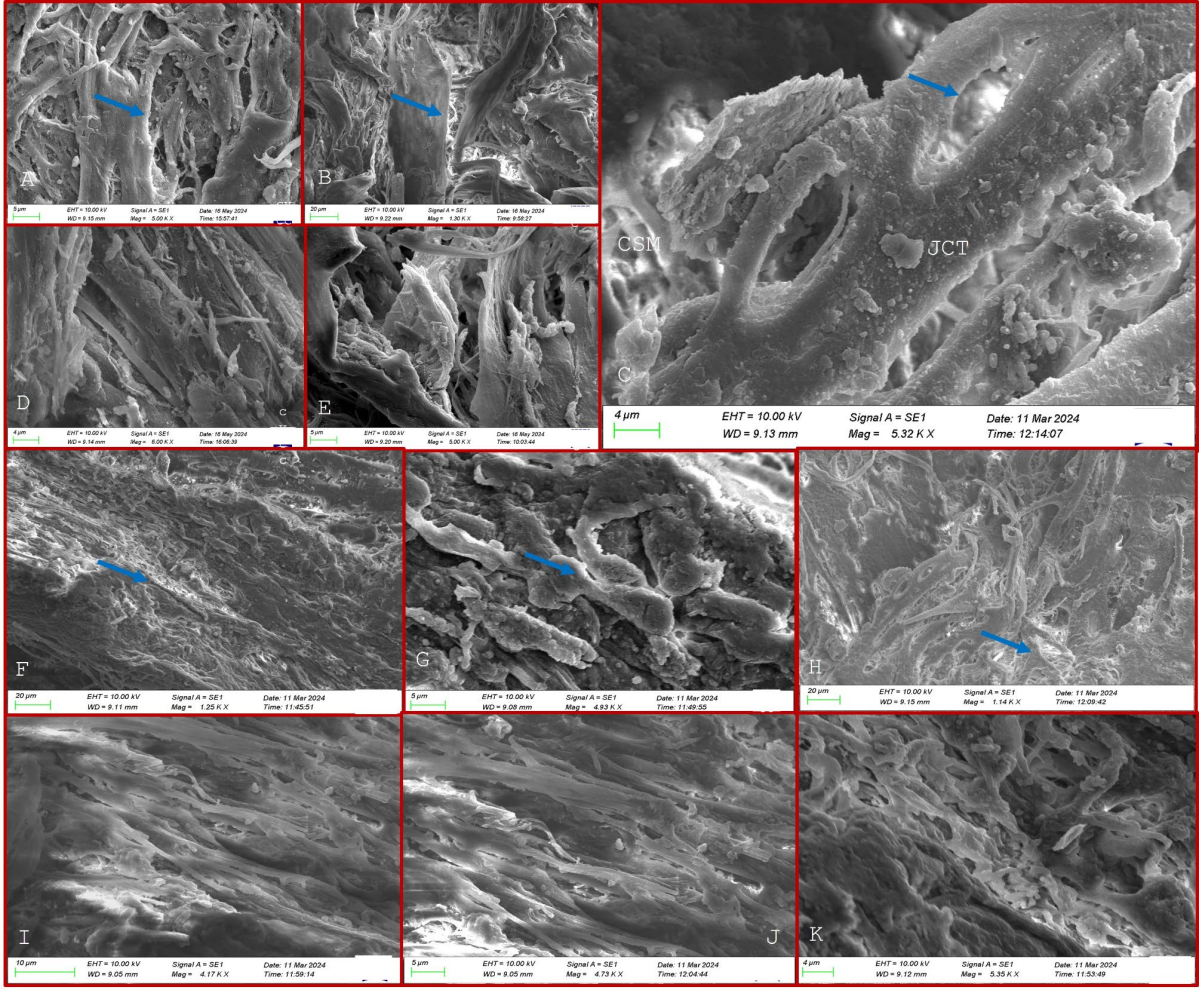
(**A**–**K**) highlights the HP and SEM features in the trabecular meshwork harvested after microincisional trabeculectomy in patients with steroid-induced glaucoma. (**A**) White glistening amorphous material is seen lining the TM beams (blue arrows) in the CSM regions and around the Schlemm’s canal opening (**C**). (**A**–**K**) shows the patchy distribution lining the TM beams-See text for full description.

On scanning electron microscopy (SEM), the TM beams in the JCT in controls appeared flat and broad with a dumbbell configuration in most areas, as shown in Fig. 1. The TM beams in the JCT region did not vary significantly morphologically between the glaucoma and control TM tissues in POAG, XFG and PACG eyes though the cellularity and the number of TM beam layers were reduced in all forms of glaucoma which was more pronounced in the JCT region, Figs. 2, 3 and 4. The TM beams in the CSM region in glaucoma were rounded and thinned in glaucoma cases, with no distinct UM region discernible on SEM in any samples.

Histopathological analysis of the TM specimens from glaucoma and controls using hematoxylin and eosin (H&E) staining revealed extremely thinned and collapsed TM beams, with a complete loss of beam width, appearing as thin cylinders with a reduced number of nucleated cells compared to controls, Figs. 1, 2, 3 and 4. These cells were mostly spindle-shaped, indicating epithelial-mesenchymal transition (EMT) of the TM cells that were seen both in the corneoscleral (CSM) meshwork and juxtacanalicular meshwork (JCT) regions. The most significant changes were observed in the CSM meshwork region, while the JCT regions showed minimal changes in TM beam morphology despite significantly reduced cellularity, Figs. 1, 2 and 3.

### Primary glaucoma

On SEM, the TM samples exhibited thinning of the TM beams across all regions, more predominantly in the CSM region, correlating with the HP features. This TM beam thinning was maximally seen in PACG, XFG, and POAG (in that order), Figs. 2 and 3. The TM beams appeared thinned and flattened, with many beams coalescing in certain places, Fig. 2. The intertrabecular (IT) spaces were widened in areas with thin beams, particularly where TM beams fused with one another in some CSM regions. The TM beams in the JCT region displayed only minimal morphological alterations in all glaucoma forms that were similar morphologically to the control TM.

### Steroid glaucoma

Both the CSM and JCT were lined with a white amorphous material that appeared to be glistening and was seen lining the borders of the TM beams in a patchy distribution, Fig. 4. In some areas, the corneoscleral meshwork was thinned, even in the absence of the material. The material was also observed lining the spaces between the TM beams, the intertrabecular spaces. In the juxtacanalicular region, these intertrabecular spaces were obliterated in some areas, possibly due to the fusion of TM beams or the deposition of extracellular matrix material filling the spaces between TM beams.

### Pseudoexfoliation glaucoma

**Fig. 5:**
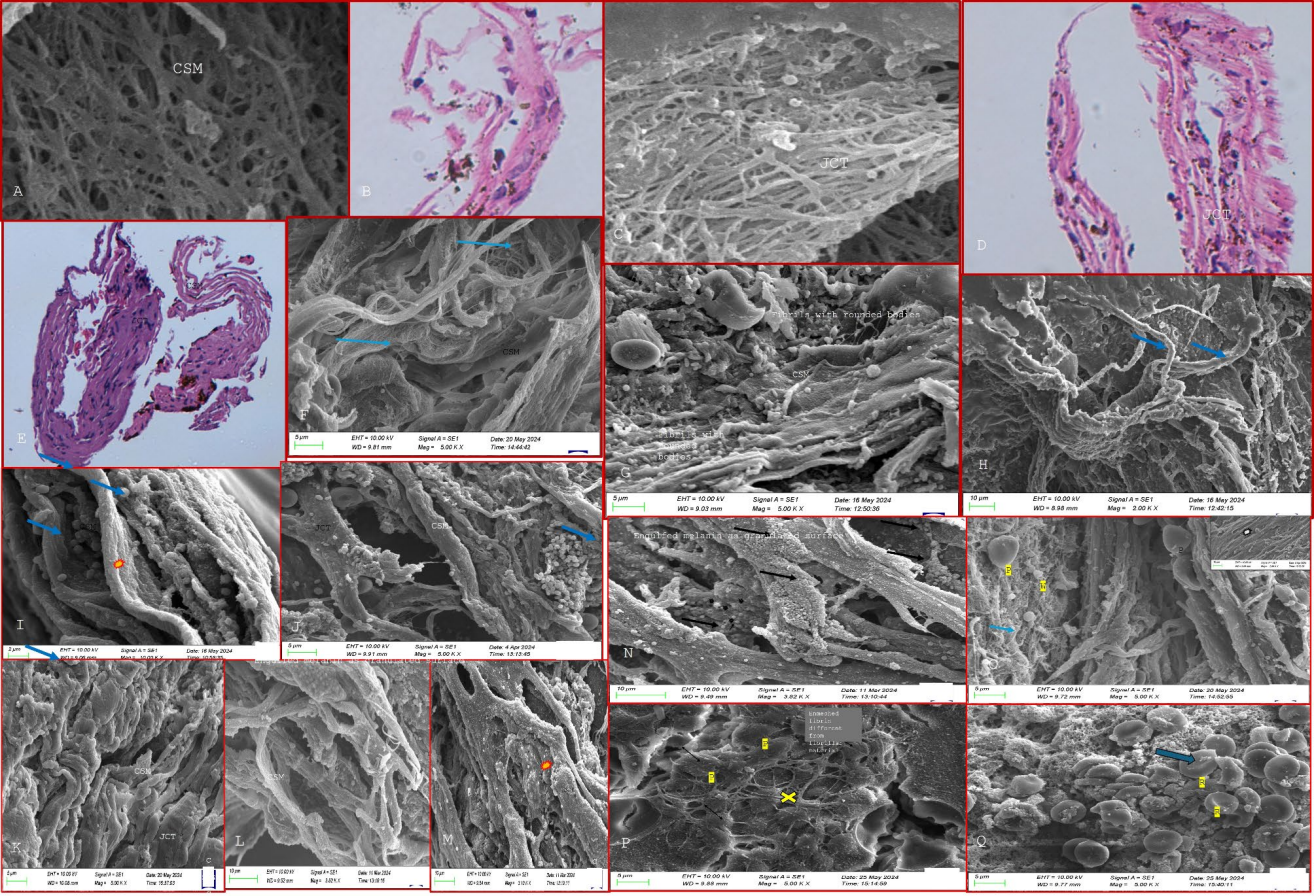
(**A**–**K**) highlights the HP and SEM features in the trabecular meshwork harvested after microincisional trabeculectomy in patients in pseudoexfoliation glaucoma- (**A**–**F**) show the thinned TM beams in the CSM region that appear as thin disrupted cylindrical beams on SEM (**A**,** C**, **E**) and HP (**B**, **D**, **F**). The JCT region seems relatively spared with relatively good cellularity and normal thick TM beams (**E**, **D**). (**I**–**O**) show the presence of fibrils with rounded bodies (**G**, **H**) with regular surface deposited on the TM beams in clusters (blue arrow) both in the CSM and JCT region. Some TM beams show melanin engulfed (black arrows) that confers a granulated appearance. P and Q show the typical appearance of platelets (P), small white blood cells (W) and biconcave red blood cells (R) in a blood clot (**C**,** D**) showing all the above enmeshed with a fibrin network (yellow cross and arrow) that are morphologically distinct from the rounded bodies or fibrin seen in cases (**I**–**O**).

While the thinning of TM beams was prominent in the CMS region, the JCT region seemed relatively spared with flat thick beams and normal cellularity on HP, Fig. 5. The intertrabecular spaces were widened in the area of TM beam thinning while it was reduced in some regions where TM beams were fused, Fig. 5. Within the CSM and JCT regions, the TM beams contained rounded bodies of variable sizes in patchy distributions with deposition of white fibrillar material at some places. These round bodies were variable in size than white blood cells (WBCs), and distinct from red blood cells (RBCs) or platelets, Fig. 5. These bodies were predominantly found in clusters or lumps spread diffusely throughout the TM sample more pronounced in regions where fibrillar material was found over the TM beams, without the surface irregularities that are characteristic of WBCs. Such bodies were however not observed in any control samples, Figs. 1 and 5. Morphologically, these bodies also differed from platelet clumps, which are typically more compact, consist of activated platelets that are flatter, and have an irregular shape, Fig. 5. Platelet clumps are usually enmeshed within fibrin along with trapped WBCs and RBCs. In some places, the TM beams had a granulated surface owing to engulfed melanin or pigment, Fig. 5, which was not seen in many regions.

## Discussion

While thinning of TM beams in the CSM regions was characteristic of all forms of primary and steroid glaucoma, the beams in the JCT regions did not display structural/morphological alterations despite reduced cellularity in that region. In XFG, rounded bodies in aggregates and clumps were found throughout the TM in eyes with XFG notably more in the CSM and JCT regions, which were distinct from platelets or white blood cells that were absent in control samples. Electron-dense material lining the TM beams were seen in patients with steroid-induced glaucoma, which may indicate GAG aggregates mostly in the CSM and JCT regions.

The distribution of rounded bodies in XFG on SEM matched the known distribution of fibrillar exfoliative material reported in XFG eyes^4,13–16^. Previous studies using transmission electron microscopy (TEM) on postmortem eyes have described fibrillar material as protein complex aggregates deposited between TM cells in the JCT and CSM regions irrespective of the presence of glaucoma^15^. The rounded bodies may represent exfoliative fibrillar material or other degraded material packaged into vesicles. The breakdown and trafficking of exfoliative material in the TM in XFG is known to involve the unfolded protein response (UPR) pathway that may recruit mechanisms for vesicular transport out of TM cells that warrants further investigation with TEM or an in-vitro study. This study used ex-vivo TM tissues from severity-matched patients with XFG, unlike previous studies that used postmortem tissues, and demonstrates the presence of vesicular bodies alongside fibrillar material that may represent an active “trafficking” that would have been absent in postmortem tissues. Further, the TM structural changes were minimal in the JCT region despite reduced cellularity suggesting preferential mechanisms of damage across different regions of the TM tissue in XFG.

The absence of structural changes despite reduced cellularity in the JCT region suggests that this region is preserved until the late stages of the disease, given its critical importance for TM physiology^1,4,7,14^. Reduced cellularity is a known feature in ageing tissues, including the TM^1,2,5,7^. Changes like TM beam thinning, collapse, and fusion of TM beams have been reported scantly in PACG eyes or enucleated trabeculectomy specimens^4,6^. Yet, none of the prior studies correlate the findings with HP sections nor analyze the role of JCT in the dissected specimens. Contrary to the traditional wisdom of the JCT region as the most involved structure and the primary site for fibrillar material deposition and TM damage in all forms of glaucoma, this study using SEM and HP analysis revealed that the most significant presence of these changes occurs within the CSM area of the TM. The TM in SIG patients showed an amorphous material lining the TM beams that may represent GAG’s reported in the literature^17–22^. An increase in nuclear size, an apparent increase in fusion vesicles arranged in linear rows at the surface of the TM cell membrane, an increase in endoplasmic reticulum in SIG, and increased fibroblastic transformation have been reported in SIG using transmission electron microscopy in-vitro in cell cultures treated with dexamethasone^19–22^. This study only found an amorphous material lining the TM beams that were thinned even in areas where these deposits were absent suggesting a global change in TM function in SIG while the GAG’s originally described as fusion vesicles onto the cell membranes suggesting part of a degradative process for eliminating these excess GAG’s. While this study only depicts the structural changes, functional damage by the reduced cellularity in aeging TM or in glaucoma types needs to be demonstrated using cell-specific signatures or TEM. Nevertheless, the correlation of findings HPE and SEM confirming the above suggests that the JCT region is the last to be involved in primary glaucoma while inducing a global change in ISG or secondary glaucoma. This may be nature’s way of protecting the most important functional region in the TM.

We did not find the UM in our samples both in HP sections or SEM since MIT procures the TM without its attachment to the scleral spur or Schwalbe’s line. The UM has been described anteriorly and posteriorly as thick cylindrical fibres that run perpendicular to Schwalbe’s ring or the peripheral cornea^1,3,4^. Since we dissected these fibers to sever the trabecular meshwork attachment from the scleral spur and the iris root posteriorly, as well as from the Schwalbe line anteriorly, it became challenging to distinguish the uveal meshwork beams in our samples. Amorphous material and fused trabeculae have been described in PACG eyes on SEM though this study did not report changes in the CSM region or the JXT since these samples were analysed only for the intracameral side of trabeculectomy specimens.^4,7^ These amorphous deposits that were seen in our samples in XFG or SIG are like the material described and were distinct from fibrinous membranes or other artifacts described on SEM in the literature^4,7,9,10,14^.

This was a qualitative study design owing to challenges in quantitative measurements on ex-vivo samples using SEM. Further, we sampled only the nasal TM region and did not study differences in TM from other regions of the eye. We did not evaluate TEM on any of our samples nor did we analyze the immunotyping or nature of the amorphous deposits in SIG or XFG. Caution is advised when interpreting the results of this study, as generalizing the findings to the entire trabecular meshwork (TM) region may be misleading; the study specifically examined TM changes only in the nasal region and does not necessarily reflect the changes in other quadrants of the eye. We correlated the changes with the histopathological sections for a direct correlation to differentiate artifacts on SEM that had not been done previously. However, we did not compare the regional signatures by immunolabelling in this essential study to confirm differential involvement and damage mechanisms in primary or secondary glaucoma. Nevertheless, this study confirms specific changes in the nasal TM ultrastructural regions by SEM correlated by HP analysis in the CSM and JCT regions in primary and secondary glaucoma. Further studies using TEM or immunolabelling techniques may indicate specific cellular signatures in TM regions that are typical for each form of glaucoma.

## Electronic supplementary material

Below is the link to the electronic supplementary material.

Supplementary Material 1

Supplementary Material 2

## Data Availability

All data generated or analysed during this study are included in this published article [and its supplementary information files]. Any photo in better resolution will be made freely available on request to the corresponding author. (Aparna Rao -aparna@lvpei.org).
