# Supplementary material for: Trabecular meshwork ultrastructural changes in primary and secondary glaucoma
Source: Sci Rep. 2025 Jan 2;15:138. doi: 10.1038/s41598-024-83834-1 (PMC11695818; doi:10.1038/s41598-024-83834-1)
Supplement: Supplementary file 1 — Supplementary Material 1 [file 41598_2024_83834_MOESM1_ESM.docx]

Table S1: Characteristics of cadaver donor corneoscleral tissue from where Trabecular meshwork was procured

| Variables (n=10) | Mean± standard deviation |
| --- | --- |
| Age (years) | 65±8.7 |
| Gender | 9 Males, 1 female |
| Systemic diseases | Diabetes mellitus-4 |
