# Supplementary figures and images for: Trabecular meshwork ultrastructural changes in primary and secondary glaucoma
Source: Sci Rep. 2025 Jan 2;15:138. doi: 10.1038/s41598-024-83834-1 (PMC11695818; doi:10.1038/s41598-024-83834-1)

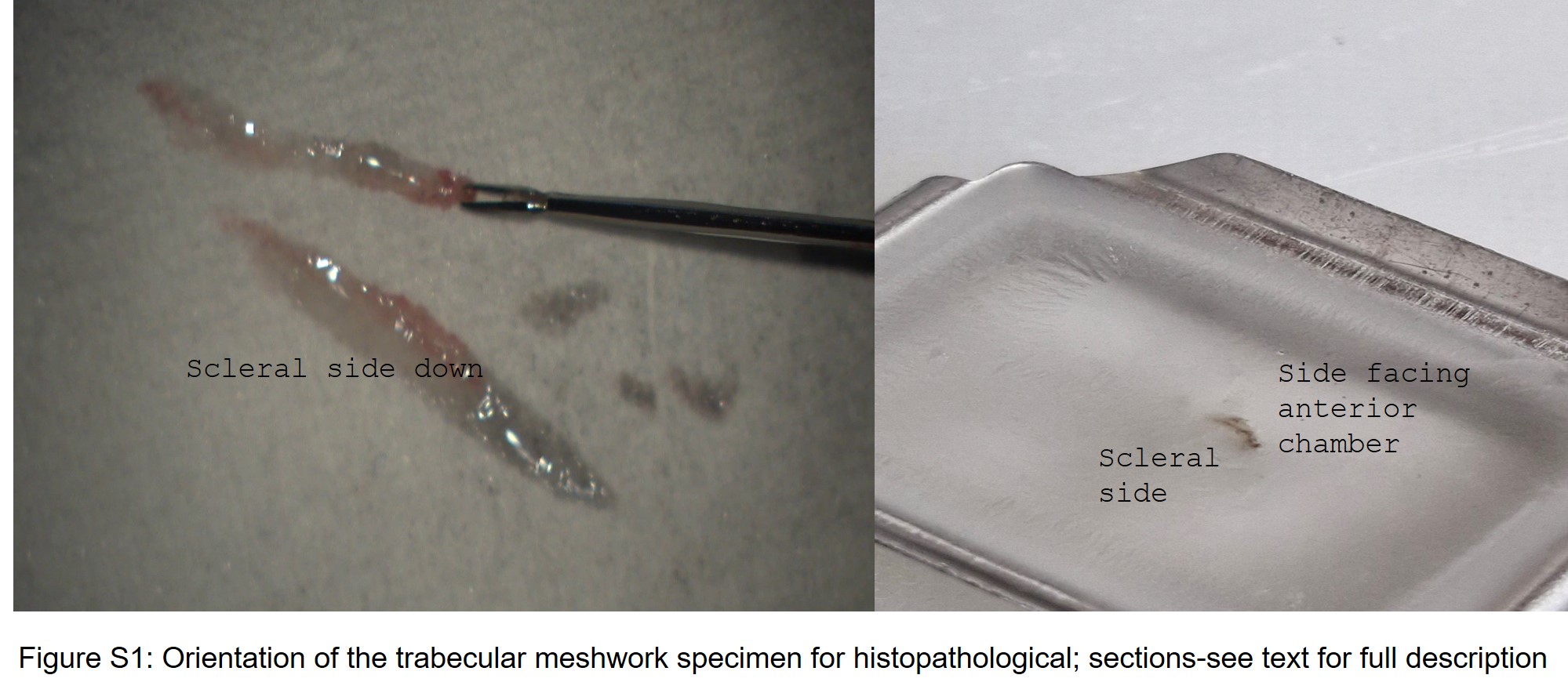

Supplement: Supplementary file 2 — Supplementary Material 2 [file 41598_2024_83834_MOESM2_ESM.jpg]
